# Supplementary Zeeman Data for the First Spectrum of Ruthenium (Ru I)

**DOI:** 10.6028/jres.063A.018

**Published:** 1959-12-01

**Authors:** J. Rand McNally, K. G. Kessler

## Abstract

Zeeman data are listed for 207 lines of Ru I between 2400 and 5400 A, all of which have been classified. The spectrograms were made at the Massachusetts Institute of Technology and were analyzed there and at the National Bureau of Standards.

Harrison and McNally^2^ published Zeeman data for 450 lines of the first spectrum of ruthenium (Ru I) in 1940. In addition unresolved patterns were measured by McNally for 175 lines, but these have never been published.^3^ Further Zeeman observations were made in 1949 by Meggers with the Bitter magnet at the Massachusetts Institute of Technology. These plates were measured by Kessler in the course of preparing material for the preceding paper. Zeeman data for 32 additional lines obtained from this last set of observations, together with the unpublished material from McNally’s thesis for 175 lines are listed below.

The observations were all made with electrodes of 1 part ruthenium powder mixed with 5 parts of silver powder. The experimental conditions and techniques used are fully described by Harrison and McNally.^2^

The wavelengths (in air) of these lines are given in column 1 of table 1. The observed *g*-value for the lower energy level involved in the transition producing the line is given in column 2, and that for the upper level is given in column 3. The complete designations for these lines are given in the preceding paper.

In the case of unresolved patterns of classified lines, where a *g*-value is known for one of the energy levels, the other *g*-value can be calculated from the separation of the strongest “*n*” components when the “*J*-values are unequal, or from the separation of the *p*” components when the *J*-values are equal. The *g*-values derived in this way are designated “*n*” or “*p*” to indicate which set of components was used. The known observed *g*-value that was used in the calculation is shown in parentheses.

The entry “Mc” in column 4 indicates that the data are taken from McNally’s thesis,^3^ and “K” denotes *g*-values determined at NBS.

## Figures and Tables

**Table 1:** 

Wavelength	Lower level	Upper level	Source
			
*A*	*Obs. g*	*Obs. g*	
2392.425	0.005	0.388	Mc
2464.699	1.49	2.02	K
2467.576	1.21	1.21	K
2476.869	1.532	1.255	K
2496.56	1.429	1.134	K
2501.885	(1.089)	1.029 *n*	Mc
2544.22	1.31	1.31	K
2558.540	(1.196)	1.24 *n*	K
2560.265	(1.624)	1.322 *p*	Mc
2567.893	(1.284)	1.541 *p*	Mc
2592.022	1.624	1.372	K
2593.700	1.033	1.092	Mc
2605.347	1.428	1.022	K
2605.853	1.07	1.06	K
2611.045	1.22	1.62	K
2651.839	1.267	1.485	K
2702.833	1.183	1.454	K
2721.562	1.248	1.473	K
2730.932	1.066	1.470	K
2735.727	(1.397)	1.474 *n*	Mc
2754.603	(1.089)	1.552 *p*	Mc
2810.029	1.269	1.462	Mc
2817.092	0.998	1.560	K
2840.537	(1.284)	1.198 *p*	Mc
2891.645	(1.16)	1.16 *n*	Mc
2913.163	1.71	0/0 *n*	Mc
2914.294	0/0	1.115	Mc
2915.614	(1.190)	1.030 *p*	Mc
2916.251	(1.35)	1.35 *n*	Mc
2917.32	(1.684)	1.115 *p*	Mc
2920.949	0/0	0.440	K
2928.487	(1.684)	1.084 *p*	Mc
2936.005	(1.232)	1.527 *p*	Mc
2937.336	(1.795)	1.533 *p*	Mc
2939.676	(1.086)	0.923 *p*	Mc
2950.532	(1.041)	1.169 *p*	Mc
2955.348	(0.757)	1.163 *p*	Mc
2993.273	1.218	1.298	Mc
3008.797	(1.25)	1.25 *n*	Mc
3013.354	(1.196)	1.234 *n*	K
3057.353	1.239	1.192	Mc
3064.834	(1.255)	1.205 *n*	Mc
3077.542	(1.534)	1.528 *n*	Mc
3084.521	1.315	1.116	Mc
3096.565	(1.086)	1.073	Mc
3105.278	1.164	0.702	Mc
3118.065	0.72	1.16	Mc
3129.835	0.000	2.383	Mc
3132.874	1.550	1.029	Mc
3144.265	(0.992)	1.022 *n*	Mc
3153.831	1.561	1.309	Mc
3170.088	(1.190)	1.272 *p*	Mc
3174.128	1.251	1.020	Mc
3179.025	(1.175)	1.143 *n*	Mc
3193.509	1.007	1.094	Mc
3250.002	(1.447)	1.108 *p*	Mc
3251.893	0.76	0.76 *n*	Mc
3277.564	(0.992)	1.059	Mc
3306.179	(1.624)	1.702 *n*	Mc
3324.999	0.008	1.019	K
3341.090	1.440	1.015	K
3345.316	(1.447)	1.393 *n*	Mc
3348.704	(1.349)	1.207 *p*	Mc
3351.930	(1.089)	1.716 *p*	Mc
3356.201	0.70	0.70 *n*	Mc
3364.100	1.08	1.08	K
3378.034	(1.397)	1.374 *n*	Mc
3385.161	1.194	1.194 *n*	Mc
3390.899	(1.175)	1.078 *p*	Mc
3414.641	(1.447)	1.434 *n*	Mc
3428.319	1.404	1.462	Mc
3443.153	(1.196)	1.464 *p*	Mc
3446.670	(1.175)	1.118 *n*	Mc
3455.385	1.19	0.45	K
3456.621	1.14	0.92	K
3459.585	(1.624)	1.446	Mc
3467.051	0.754	1.020	Mc
3494.254	1.250	1.452	K
3498.944	(1.397)	1.379 *n*	Mc
3502.418	(1.420)	1.460 *n*	Mc
3537.941	(1.249)	1.269 *n*	Mc
3587.204	1.036	1.036 *n*	Mc
3601.487	1.041	1.220	Mc
3625.197	(1.534)	1.558 *n*	Mc
3626.740	0.840	0.919	Mc
3672.059	(0.834)	1.303 *p*	Mc
3672.378	(1.684)	1.717 *n*	Mc
3676.952	(1.175)	1.415 *p*	Mc
3701.312	0.000	0/0 *n*	Mc
3738.914	1.029	0.852	Mc
3742.798	(1.164)	1.146 *n*	Mc
3746.218	1.28	1.28 *n*	Mc
3781.171	1.006	1.030	Mc
3786.065	(1.000)	0.956 *n*	Mc
3803.191	0.923	1.053	Mc
3814.848	1.14	1.21	Mc
3817.293	(1.164)	1.189 *n*	Mc
3835.983	1.186	1.540	Mc
3838.069	(1.563)	1.526 *n*	Mc
3850.441	1.45	1.45 *n*	Mc
3857.551	(0.992)	1.012 *n*	Mc
3862.690	(0.834)	0.853 *n*	Mc
3864.851	0/0	0.78 *n*	Mc
3905.993	(1.343)	1.061 *p*	Mc
3923.486	1.033	1.048	Mc
3924.636	(1.007)	1.07 *p*	K
3937.919	1.53	1.48	Mc
3941.672	(1.315)	1.711 *p*	Mc
3950.041	(1.007)	0.962 *n*	Mc
3952.290	(0.676)	1.006	Mc
3974.504	(1.420)	1.470	Mc
4005.089	(1.175)	1.004 *p*	Mc
4014.153	0.69	0.69	K
4026.492	(1.070)	0.703	K
4028.434	(1.086)	1.358 *p*	Mc
4040.474	0.997	1.055	K
4046.883	0.693	1.058	K
4062.854	(1.007)	0.926 *p*	Mc
4076.730	1.233	0.524	K
4108.055	(1.190)	1.487 *p*	Mc
4134.854	(1.684)	1.066 *p*	Mc
4156.254	(1.007)	1.035 *p*	Mc
4159.168	(1.041)	1.036 *n*	Mc
4175.436	(1.007)	1.219 *p*	Mc
4182.455	(1.175)	1.137 *n*	Mc
4185.465	1.08	1.08	K
4239.660	(0.697)	1.081 *n*	K
4281.941	1.164	1.174	Mc
4312.494	0.71	0/0 *p*	Mc
4314.308	1.590	1.609	K
4325.059	1.327	1.438	K
4338.675	(1.343)	0.938 *p*	Mc
4340.351	(1.041)	0.895 *p*	Mc
4364.108	(1.343)	0.796 *p*	Mc
4394.970	(1.164)	1.047 *p*	Mc
4438.343	(1.162)	0.438 *p*	K
4439.745	(1.086)	1.024 *n*	Mc
4645.09–	1.005	(1.013)	Mc
4774.004	(0.927)	0.900 *n*	Mc
5133.895	(0.757)	1.029 *p*	Mc
5142.772	(1.420)	1.370 *n*	Mc
5171.026	(1.447)	1.396 *n*	Mc
5266.469	1.337	1.595	Mc
5280.812	1.426	1.426 *n*	Mc
5284.089	1.282	1.632	Mc
5377.840	1.22	1.19	Mc

## Footnotes

2 G. R. Harrison and J. R. McNally, Jr. Phys. Rev. 58, 703 (1940).

3 J. R. McNally, Jr., Thesis, Mass. Inst. Technol. (1941) unpublished.

